# *C-X-C motif chemokine 16*, modulated by *microRNA-545*, aggravates myocardial damage and affects the inflammatory responses in myocardial infarction

**DOI:** 10.1186/s40246-021-00314-7

**Published:** 2021-02-26

**Authors:** Fang-Qian Liang, Jing-Yuan Gao, Ji-Wei Liu

**Affiliations:** 1 Department of General practice, North China University of science and technology affiliated Hospital, Tangshan, 063000 Hebei People’s Republic of China; 2 Jingzhou Central Hospital, Heart function examination room, No.60 Jingzhong Road, Jingzhou District (Jingzhou ancient town flower terrace), Jingzhou, 434020 Hubei People’s Republic of China

**Keywords:** Myocardial infarction, Hypoxia/reoxygenation, Proliferation, Apoptosis, Inflammation

## Abstract

**Background:**

Myocardial infarction (MI), a common type of coronary heart disease, is the major cause of morbidity and mortality around the world. Chemokine-mediated inflammatory cell infiltration and local inflammatory damage response are recent research hotspots. Hence, we attempted to examine the role of *C-X-C motif chemokine 16* (*CXCL16*) as a potential candidate in MI.

**Methods:**

Human cardiomyocytes were treated with hypoxia/reoxygenation (H/R) to establish an in vitro cell model. GEO database provided the clinical data of MI patients and GSEA verified the relationship of chemokine and MI. CCK-8 and flow cytometry analyses were used to measure cell viability and apoptosis. Bioinformatics analysis and luciferase reporter assay were conducted to determine the correlation between *CXCL16* and *miR-545*. qRT-PCR and western blot assays were performed to investigate the expression level of the indicated genes. The activity of lactate dehydrogenase (LDH) and the levels of TNF-α, IL-6, IL-1β, and IL-10 were explored using ELISA assay.

**Results:**

CXCL16 was increased in MI. CXCL16 knockdown can reverse the inhibitory effect of H/R treatment on cell viability, while overexpression of CXCL16 showed the opposite trend. *MiR-545* directly targeted *CXCL16* and negatively regulated CXCL16 levels. *MiR-545* promoted cell proliferation and inhibited apoptosis in the MI cell model, which attenuated the CXCL16-induced injury on cardiomyocytes.

**Conclusion:**

These findings demonstrated that *CXCL16* aggravated MI damage through being directly targeted by *miR-545* and mediating inflammatory responses, thereby providing potential therapeutic targets for MI therapy.

**Supplementary Information:**

The online version contains supplementary material available at 10.1186/s40246-021-00314-7.

## Background

Myocardial infarction (MI), induced by coronary artery occlusion, is one of the leading causes of cardiac event-related mortality worldwide and finally results in ischemic death of cardiac tissue [1]. It represents a condition that myocardial perfusion is suspended, and the supply of oxygen and nutrient to the heart is arrested [2]. The pathological stages of MI mainly include three phases: inflammation, proliferation, and maturation [3]. Recently, inflammation has been widely considered as a crucial event during atherosclerosis and other heart diseases. Moreover, inflammation affects the remodeling process suffered from MI in the heart [4]. Thus, deciphering an effective therapy specially targeting inflammation would greatly improve the condition of MI patients and attenuate mortality.

Chemokine-mediated inflammatory cell infiltration and local inflammatory damage responses emerge as hotspots in recent years. Therefore, we performed Gene Set Enrichment Analysis (GSEA) to explore whether the progression of MI is linked with the chemokine signaling pathway. Chemokines are a string of small chemotactic cytokines with molecular weights of 8–12 kDa, which play a central role in the migration of leukocyte and other cellular motilities [5]. Interestingly, the migration of leukocyte into the vessel wall induced by chemokine is considered to be an early event in atherosclerosis development, as well as the major cause of MI [6]. Song et al. indicated that the increasing mesenchymal stem cell platelet in peripheral blood of MI patients is associated with the *CXCR4/SDF-1* axis [7]. The potential effect of *CXCL9* in MI has been investigated by Lin et al., which suggested that upregulation of CXCL9 in MI might exert an important role in post-MI cardiac fibrosis via activating cardiac fibroblasts [8]. Chrono-pharmacological targeting *CCL2/CCR2* axis can ameliorate atherosclerosis [9]. Among the currently known chemokines, CXCL16 is somewhat peculiar with a glycosylated mucin-like stalk, and sensitive to a disintegrin and metalloprotease [10]. CXCL16 is expressed in both transmembrane and soluble forms [11]. CXCL16 can chemo attract immune cells to the inflammatory sites and act as the scavenger receptor for oxidized lipoproteins, which manifest its potential crucial role in MI [12]. Notably, in a soluble form, CXCL16 has been identified as an independent factor of cardiovascular death and morbidity in acute coronary syndromes [13]. However, the biological functions of soluble CXCL16 in MI and the underlying mechanism have not been fully illustrated.

*MiRNAs* are a class of endogenous and non-coding small single-stranded RNAs and could bind with the 3′ untranslated regions of target genes to regulate the translation process. Emerging evidences have shown that *miRNAs* are implicated in multiple biological processes and also act as important biomarkers in cardiovascular diseases, including MI. For example, *miR-208a* elevates the expression of myocardial endoglin and promotes myocardial fibrosis in acute MI [14]. *miR-98* inhibits the MI-induced apoptosis through downregulating caspase-3 [15]. *miR-135b* prevents cardiomyocytes from infarction via restraining the NLRP3/caspase-1 signaling pathway [16]. The role of *miR-545* in human disease has attracted continuous attention. *miR-545* increases the replication of enterovirus 71 by targeting phosphatase and tumor necrosis factor receptor-associated factor 6 [17]. *miR-545* is reduced in glioma cells, which may correlate with the upregulation of *circPRKCI*. Targeting *circPRKCI/miR-545* cascade might efficiently suppress human glioma cells [18]. Additionally, *miR-545* could promote cell proliferation via targeting RIG-I or MT1M in hepatocellular carcinoma [19, 20]. However, the effect of *miR-545* on the progression of MI remains unknown.

In this present report, the effect of CXCL16 on the H/R-treated human neonatal cardiomyocytes was evaluated by using loss/gain-of-function assays. The regulatory role of *miR-545* on CXCL16 expression, as well as their interactions, was investigated to further reveal the potential mechanisms. In addition, the levels of inflammatory cytokines were detected to identify whether CXCL16 is implicated in the inflammatory responses post-MI.

## Methods

### Data analysis

Gene Expression Omnibus (GEO) database was accessed to download the corresponding data sets for CXCL16 (GSE60993) and *miR-545* (GSE24548) expression in MI, respectively. The data set GSE60993 was collected from peripheral blood containing 17 patients and 7 control samples, while *miRNA* expression profiling of GSE24548 was analyzed in platelets from 4 patients with first acute MI and in 3 controls. The differentially expressed genes (DEGs) or miRNAs (DEmiRs) between patients and controls were analyzed by GEO online analysis tool GEO2R. The criteria were |log fold change (FC)| ≥ 1 and *P* < 0.05. Gene set enrichment analysis (GSEA) was utilized to verify the correlation between chemokine and MI.

### Cell culture and establishment of the H/R in vitro model

Human neonatal cardiomyocytes (cat. no: 36044-21-T150; CELOROGEN, USA) were incubated in Dulbecco’s modified Eagle’s medium (DMEM) supplemented with 10% fetal bovine serum (FBS), 100 U/mL penicillin, and 100 μg/mL streptomycin at 37 °C with 5% CO_2_. To mimic the hypoxia/reoxygenation (H/R) condition, cells were firstly incubated under a hypoxic environment (94% N_2_, 5% CO_2_, and 1% O_2_) at 37 °C for 6 h and then reperfused in a normoxic room for 24 h.

### Transfection

To regulate the expression of CXCL16, pcDNA3.1-CXCL16 (4 μg) and si-CXCL16 (100 pmol) were synthesized by GenePharma (Shanghai, China). Meanwhile, pcDNA3.1-empty vector (vector, 4 μg) and non-targeting siRNA (si-con, 100 pmol) were considered as internal controls. *MiR-545 mimic* (100 pmol), *miR-545 inhibitor* (100 pmol), and corresponding negative controls (blank group) were purchased from GenePharma (Shanghai, China) to elevate or decrease the expression of *miR-545*. These agents were transfected into cardiomyocytes using Lipofectamine 3000 reagent (Invitrogen, Carlsbad, CA, USA) according to the manufacturers’ protocols. Transfected cells were collected for further experiments after 24 h transfection.

### Cell counting kit 8 analysis

After 24 h post-transfection, cells (1000 cells/100 μL suspension) were inoculated into a 96-well plate and cultured under conditions of 37 °C with 5% CO_2_. After H/R treatment, 10 μL of CCK-8 solution was added into each well and cultivated with these cells for 1.5 h at 37 °C and 5% CO_2_. The optical density (OD) value was measured at 450 nm under the microplate reader (Infinite M200, TECAN).

### Dual-luciferase reporter gene assay

The fragments harboring the putative *miR-545* binding site were amplified and cloned into pmiR-RB-REPORTTM vector (0.2 μg; Promega, USA) to construct wild type CXCL16 3′-UTR (wt-CXCL16) or mutant CXCL16 3′-UTR (mut-CXCL16) luciferase reporter vectors. Then, luciferase reporter constructs and *miR-545 mimic* (50 pmol) or *miR-545 inhibitor* (50 pmol) were co-transfected into cells by Lipofectamine 3000 (Invitrogen) for 48 h. Luciferase activity was detected by using Dual-Luciferase Reporter Assay Kit (Promega).

### Annexin V-FITC/propidium iodide (AV/PI) staining

Cells were collected at 24 h after transfection. Next, transfected cells were exposed to the H/R condition. Afterwards, cells were suspended to the final density of 1–5 × 10^6^/mL using pre-cold PBS and 1× binding buffer. Following staining with Annexin V-FITC and PI in the dark, cell apoptosis capability was examined by flow cytometry and analyzed using Flowjo software.

### QRT-PCR

TRIzol solution (Invitrogen) was utilized to extract total RNA from cells. PrimeScript RT Reagent Kit (Takara, Japan) and SYBR Premix Ex Taq II (TaKaRa) were employed to synthesize cDNA through reverse transcriptions and performed real-time PCR on 7900HT real-time PCR system to measure the expression of CXCL16.

The cDNA of *miR-545* was reversed transcribed with the aid of MiScript Reverse Transcription kit (Qiagen). Real-time PCR was performed using MiScript SYBR-Green PCR kit (Qiagen) to assess the expression level of *miR-545*.

The relative expression of CXCL16 and *miR-545* were normalized to GAPDH or U6. Each experiment was conducted in three times and all reactions were repeated in triplicate. The 2^−ΔΔCt^ method was used to calculate the relative expression levels and primers used in this investigation are as follows:

*miR-545*:F: 5′-TCA GCA AAC ATT TAT TGT GTG C-3′

R: universal primer for the kit;

U6: F: 5′-CTC GCT TCG GCA GCA CA-3′

R: universal primer for the kit;

CXCL16: F: 5′-AAG CCA TTG AGA CAC CAG CTG-3′,

R: 5′-ACC TCG CTC TGA CTC CCA GA-3′;

GAPDH: F: 5′-GTC TCC TCT GAC TTC AAC AGC G-3′,

R: 5′-ACC ACC CTG TTG CTG TAG CCA A-3′.

### Western blot analysis

After 48 h transfection, total proteins of cells were isolated with radio immunoprecipitation (RIPA) buffer containing phenylmethyl sulfonylfluoride (PMSF, protease inhibitor) and quantified by BCA method. Subsequently, isolated proteins were denatured at 95 °C for 5 min and separated in 12% SDS-PAGE. Next, the separated proteins were transferred onto the PVDF membranes. The 5% non-fat milk was utilized to block PVDF membranes for 1 h at room temperature and indicated primary antibodies were then used to incubate PVDF membranes overnight at 4 °C against CXCL16, PCNA, and GAPDH. Following washed by TBST three times, PVDF membranes were reacted using corresponding secondary antibody at room temperature for 1 h. The protein bands were observed by enhanced chemiluminescence (ECL) and quantified with QUANTITY ONE software.

### Enzyme-linked immunosorbent (ELISA) experiment

Following specific transfections and H/R stimulation, cell culture supernatant was harvested for ELISA assay. The lactate dehydrogenase (LDH; Nanjing Jiancheng Bioengineering, Nanjing, China) and ELISA (Roche, Germany) were used to explore the activity of LDH and levels of TNF-α, IL-6, IL-1β, and IL-10 as the protocols of manufacturers.

### Statistical analysis

GraphPad Prism 5.0 and SPSS 22.0 software were applied for all statistical analysis. Differences of two groups were determined by Student’s *t* test, and comparisons in multiple groups were analyzed using one-way ANOVA followed by Tukey’s post hoc test. All data were exhibited as mean ± standard deviation (SD). *P* < 0.05 was considered as significantly significant.

## Results

### CXCL16 expresses at higher levels in MI and human neonatal cardiomyocytes in response to H/R treatment

**Fig. 1:**
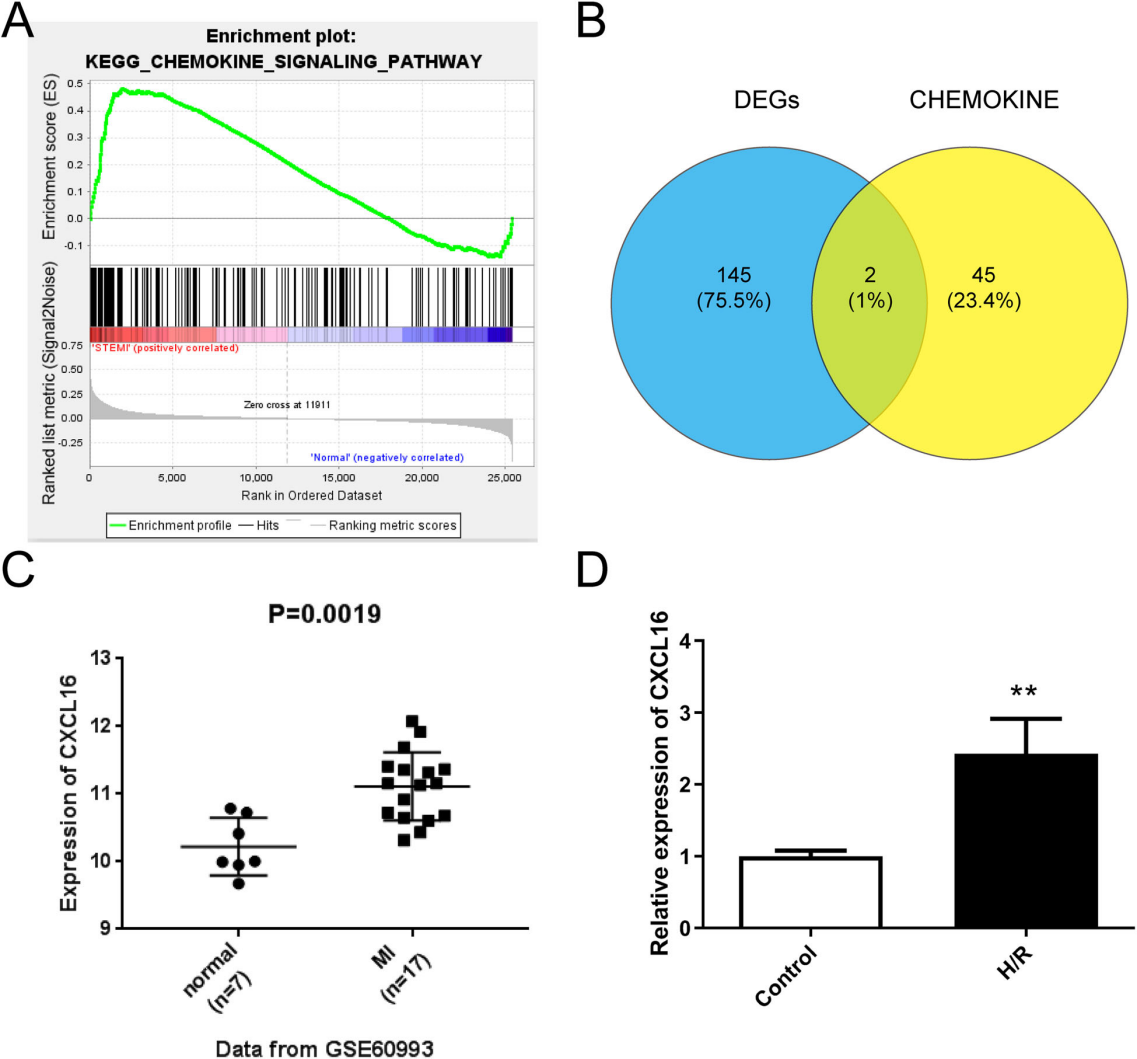
CXCL16 expression was elevated in MI and human neonatal cardiomyocytes in response to H/R treatment. **a** GSEA analysis was applied to detect the correction between chemokine-related genes and MI. **b** The Venn curve exhibited that there are two overlapped differentially expressed genes: CXCL16 and NCF1. Blue represents the DEGs obtained from the GEO database and yellow represents the upregulated chemokine genes in MI. **c** Relative expression of CXCL16 in MI tissues (*n* = 17) and normal specimens (*n* = 7) based on the GSE60993, *P* = 0.0019. **d** Relative expression of CXCL16 in human neonatal cardiomyocytes evoked by the H/R and control group, **P < 0.01 *vs.* control. *n* = 3 independent experiments. All data were exhibited as mean ± standard deviation (SD)

The appearance of chemokines is the main feature of inflammatory response after myocardial injury [5]. Thus, in order to investigate the relationship of chemokine and MI, GSEA was implemented based on the KEGG gene set of MSigDB database and revealed that chemokines were positively correlated with the MI process (*P* = 0.016, Fig. 1a) (Table S1). Then, we interacted the chemokine-related genes (*n* = 47) (Table S2) derived from GSEA with differentially expressed genes (DEGs; *n* = 147) (Table S3) in MI obtained from the GEO database, and two common genes *CXCL16* and *NCF1* were ultimately achieved (*P* = 0.016, Fig. 1b). We finally selected *CXCL16* as the research project followed by a comprehensive analysis. GSE60993 data set showed that soluble CXCL16 was significantly increased in MI tissue specimens (*n* = 17) compared with human normal samples (*n* = 7) (*P* = 0.0019, Fig. 1c). Moreover, in the in vitro H/R cell model, the expression of CXCL16 was also increased relative to the control (**P < 0.01, Fig. 1d). These data indicated that CXCL16 might be associated with the progression of MI.

### CXCL16 inhibits cell viability in human neonatal cardiomyocytes treated with H/R

**Fig. 2:**
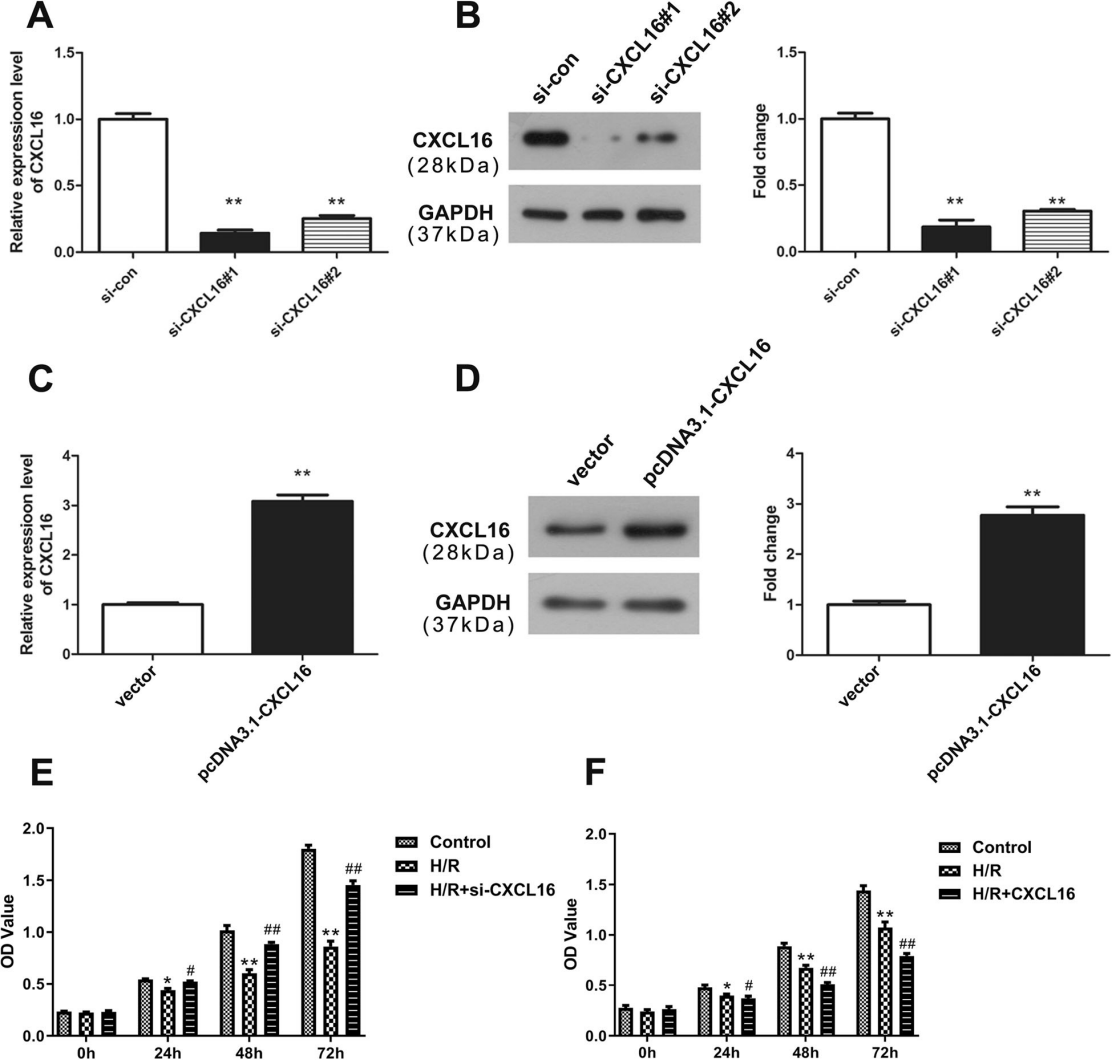
CXCL16 modulated cell viability of human neonatal cardiomyocytes treated with H/R. **a**, **b** After si-CXCL16#1 and si-CXCL16#2 transfections, CXCL16 expression was measured at both mRNA and protein levels using qRT-PCR and western blot assays, *n* = 3 independent experiments. **P < 0.01 *vs.* si-con. **c**, **d** qRT-PCR and western blot assays revealed the expression of CXCL16 after the transfection of pcDNA3.1-CXCL16, *n* = 3 independent experiments. **P < 0.01 *vs.* vector. **e**, **f** Cell viability was measured using CCK-8 analysis, *n* = 3 independent experiments. **P < 0.01 *vs.* control, ^##^*P* < 0.01 *vs.* H/R. All data were exhibited as mean ± standard deviation (SD)

Subsequently, we regulated the expression of CXCL16 to determine its biological roles in cell viability after H/R treatment. QRT-PCR and western blot analyses exhibited that CXCL16 expression was remarkably decreased in cells due to the transfection of si-CXCL16#1 and si-CXCL16#2 (**P < 0.01, Fig. 2a, b). Meanwhile, both mRNA and protein expressions of CXCL16 were overexpressed after pcDNA3.1-CXCL16 transfection (**P < 0.01, Fig. 2c, d). After H/R treatment, cell viability was significantly inhibited (**P < 0.01, Fig. 2e, f). Downregulation of CXCL16 was observed to partially rescue the cell viability of cardiomyocytes that were repressed by H/R treatment (^##^*P* < 0.01, Fig. 2e), while overexpression of CXCL16 enhanced the inhibitory effect of H/R on cell viability (^##^*P* < 0.01, Fig. 2f). Collectively, CXCL16 can inhibit cell viability in H/R-induced cardiomyocytes.

### MiR-545 directly targets CXCL16 and attenuates the expression level of CXCL16 in H/R-stimulated human neonatal cardiomyocytes

**Fig. 3:**
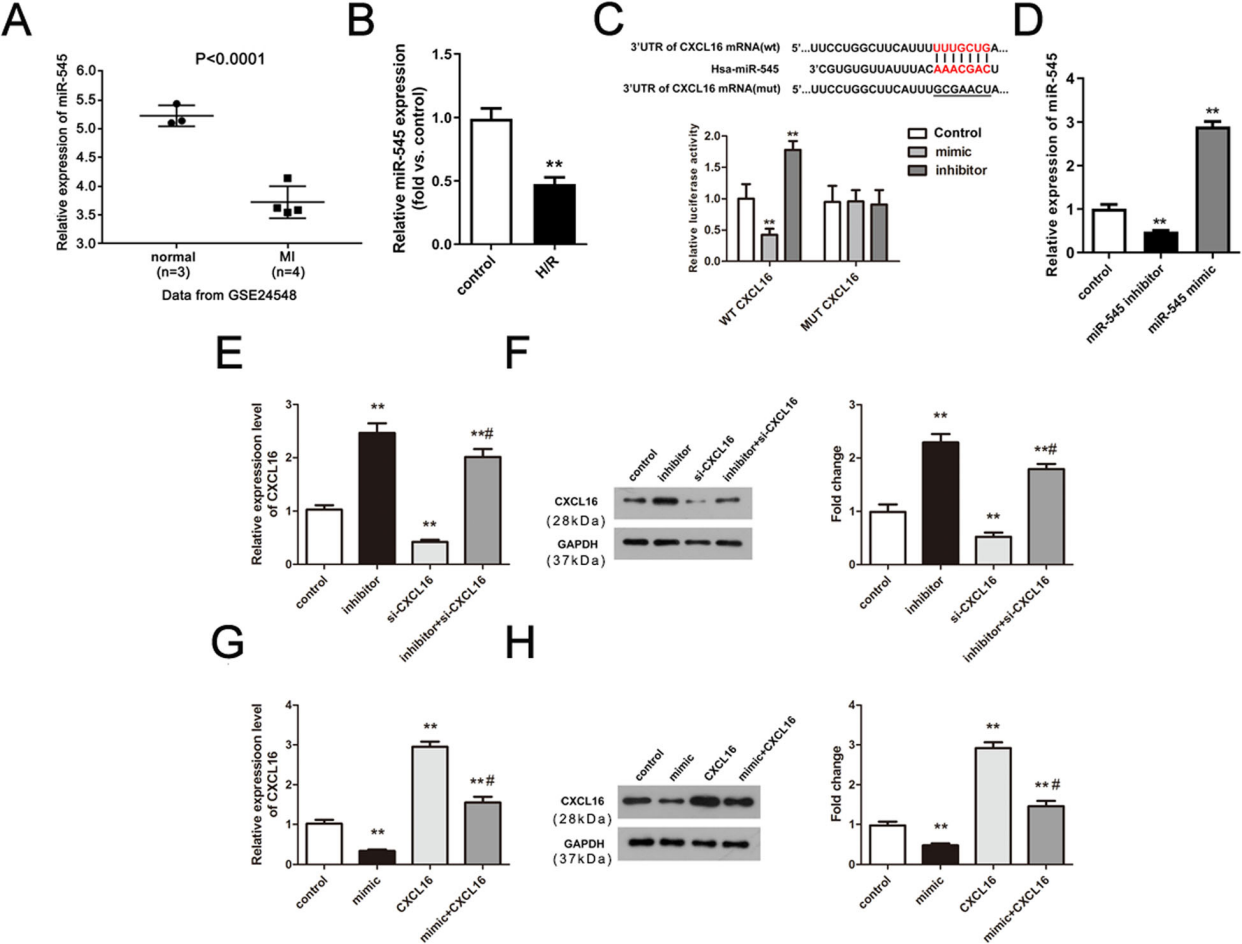
*MiR-545* can directly target CXCL16 and inhibit the expression level of CXCL16 in H/R-stimulated human neonatal cardiomyocytes. **a** Relative expression of *miR-545* in MI (*n* = 4) and control (*n* = 3) based on the GSE24548 data set, *P* < 0.0001. **b** Expression of miR-545 in vitro H/R cell model, *n* = 3 independent experiments. **P < 0.01 *vs.* control group. **c** Relative luciferase activity was determined using dual-luciferase reporter gene assay, *n* = 3 independent experiments. **P < 0.01 *vs.* negative control (NC). **d** Relative expression of *miR-545* in cardiomyocytes transfected with *miR-545 inhibitor* and *mimic*, *n* = 3 independent experiments. **P < 0.01 *vs.* control group. **e**–**h** qRT-PCR and western blot experiments were performed to examine the CXCL16 mRNA and protein expression after diverse transfection, *n* = 3 independent experiments. **P < 0.01 *vs.* control and ^##^*P* < 0.01 *vs.* si-CXCL16 or CXCL16. All data were exhibited as mean ± standard deviation (SD)

Prediction website was employed to predict the upstream *miRNAs* of *CXCL16* and a total of 37 *miRNAs* were obtained. Based on the GEO database, a total of 25 differentially expression *miRNAs* with lower expression levels was identified in MI. After intersection, *miR-545* and *miR-34a* were achieved, and *miR-545* was selected for the following research. As shown in Fig. 3a, *miR-545* was markedly decreased in MI (*P* < 0.0001). In the in vitro cell model, the expression of *miR-545* was decreased compared with the control group (**P < 0.01, Fig. 3b). The sequences of putative binding site between *miR-545* and *CXCL16* are exhibited in Fig. 3c. Compared with the negative control (NC), *miR-545 mimic* significantly reduced the luciferase activity of WT CXCL16 while *miR-545 inhibitor* elevated the luciferase activity of WT CXCL16. However, both *miR-545 mimic* and *miR-545 inhibitor* had no effects on the luciferase activity of MUT CXCL16 (**P < 0.01, Fig. 3c). Besides, *miR-545 inhibitor* significantly increased the expression level of CXCL16, whereas *miR-545 mimic* obviously inhibited CXCL16 expression (**P < 0.01, Fig. 3d–h). Furthermore, co-transfections of *miR-545 inhibitor* and si-CXCL16 or *miR-545 mimic* and CXCL16 could reverse the effects of si-CXCL16 or CXCL16 (^##^*P* < 0.01, Fig. 3d–h). In summary, *miR-545* directly targets CXCL16 and inhibits the expression level of CXCL16 in MI.

### miR-545 elevates cell proliferation and eliminates apoptosis through targeting CXCL16

**Fig. 4:**
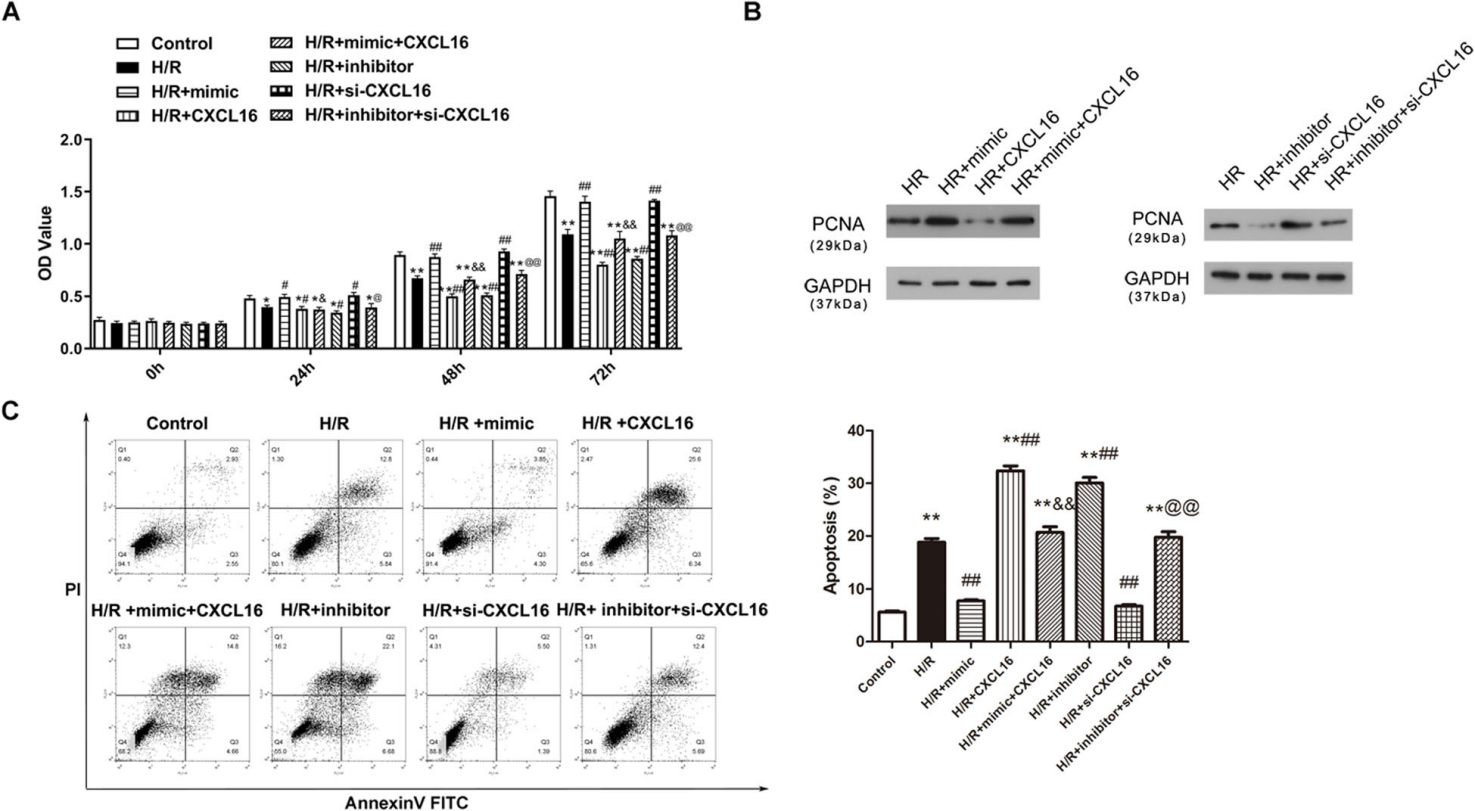
*miR-545* elevates cell proliferation and eliminates apoptosis through targeting CXCL16. Results of in vitro functional experiments demonstrated that *miR-545* was involved in the effects of CXCL16 on **a**, **b** cell viability and **c** apoptosis, *n* = 3 independent experiments. **P < 0.01 *vs.* control, ^##^*P* < 0.01 *vs.* H/R, ^&^*P* < 0.05 and ^&&^*P* < 0.01 *vs.* H/R + CXCL16, and ^@^*P* < 0.05 and ^@@^*P* < 0.01 *vs.* H/R + si-CXCL16. All data were exhibited as mean ± standard deviation (SD)

To further assess the underlying mechanism of *miR-545/CXCL16* axis in MI, CCK-8 and flow cytometry experiments were conducted for the detection of cell viability and apoptosis, respectively. Results of the CCK-8 assay showed that the H/R-treated cardiomyocyte viability was promoted after *miR-545 mimic* and si-CXCL16 transfections compared with the H/R group (*P* < 0.01, Fig. 4a). The opposite tendency was exhibited after the transfection of *miR-545 inhibitor* and high-regulated CXCL16 (*P* < 0.01, Fig. 4a). The addition of *miR-545 mimic* partially abolished the inhibitory impacts of CXCL16 on cell viability, and co-transfection of *miR-545 inhibitor* and si-CXCL16 attenuated the promoting effects of si-CXCL16 on cell viability in the H/R cell model (*P* < 0.01, Fig. 4a). As shown in Fig. 4b, in consistency with cell viability results, PCNA expression was significantly increased owing to the transfection of *miR-545 mimic* or si-CXCL-16 in H/R-induced cells, whereas CXCL16 overexpression or *miR-545* inhibition dramatically abolished PCNA. Co-transfection of *miR-545 mimic* and pcDNA3.1-CXCL16 overturned the effect of *miR-545 mimic* or pcDNA3.1-CXCL16, and the co-transfection of *miR-545 inhibitor* and si-CXCL16 reversed the role of *miR-545 inhibitor* or si-CXCL16. Furthermore, cell apoptosis showed the opposite trends with the proliferative capability after various treatments (*P* < 0.01, Fig. 4c). To sum up, these above observations indicated that *miR-545* can promote cell proliferation and inhibit apoptosis through negatively regulating CXCL16.

### MiR-545/CXCL16 axis mediates the activity of LDH and the levels of inflammatory cytokines

**Fig. 5:**
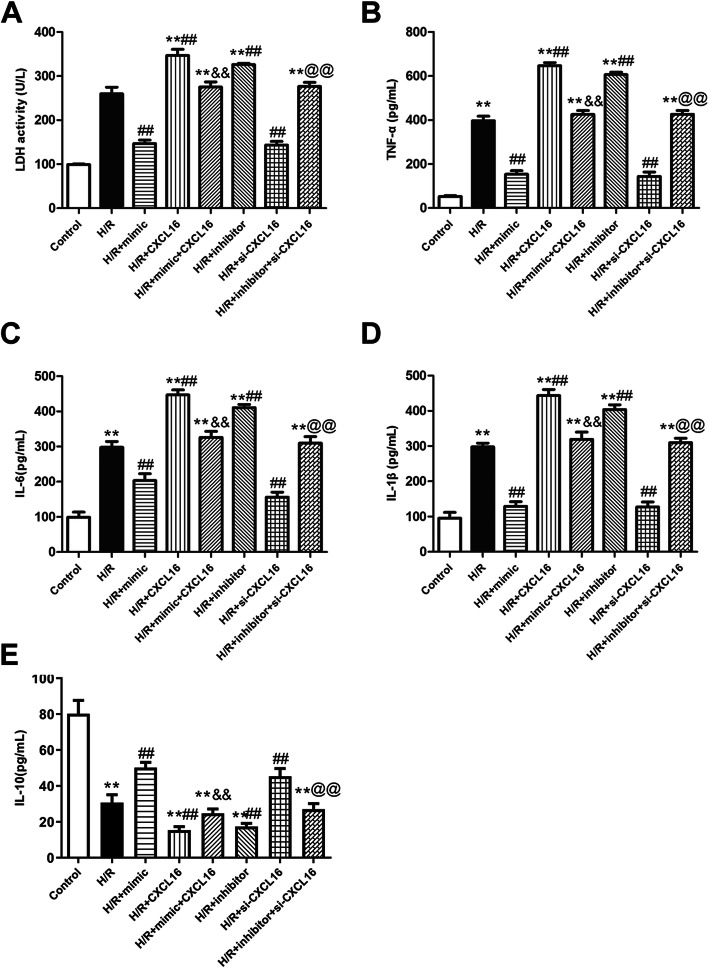
*MiR-545*/CXCL16 axis regulated the activity of LDH and inflammatory response. **a** The activity of LDH and the levels of inflammatory cytokines including **b** TNF-α, **c** IL-6, **d** IL-1β, and **f** IL-10 were assessed using ELISA analysis, *n* = 3 independent experiments. **P < 0.01 *vs.* control, ^##^*P* < 0.01 *vs.* H/R, ^&&^*P* < 0.01 *vs.* H/R + CXCL16, and ^@@^*P* < 0.01 *vs.* H/R + si-CXCL16. All data were exhibited as mean ± standard deviation (SD)

Given the important role of inflammation in MI, we next detected the activity of LDH and inflammatory cytokines for further examination of the role of *miR-545/CXCL16* axis. After H/R treatment, the activity of LDH and the levels of TNF-α, IL-6, and IL-1β were significantly increased (*P* < 0.01, Fig. 5a–d). Compared with the H/R group, they were inhibited in the H/R + mimic group and H/R + si-CXCL16 group, while they were promoted in the H/R + inhibitor group and H/R + CXCL16 group (*P* < 0.01, Fig. 5a–d). Moreover, the co-transfection of mimic and CXCL16 attenuated the promoting activity of LDH caused by H/R + mimic treatment, as well as inflammatory factors (TNF-α, IL-6, and IL-1β). Downregulation of LDH activity, TNF-α, IL-6, and IL-1β induced by H/R + si-CXCL16 were significantly elevated due to the addition of *miR-545 inhibitor* (*P* < 0.01, Fig. 5a–d). Likewise, the level of IL-10 was opposite with the above pro-inflammatory cytokines (*P* < 0.01, Fig. 5e). Therefore, we concluded that *miR-545/CXCL16* axis might modulate the development of MI through regulating inflammation.

## Discussion

The concept of cardiovascular disease is firstly proposed by Herberden in 1772 [21], while James Herrick did not define MI until 1912 according to the relationship between coronary thrombosis and coronary artery disease [22]. From then on, the research on the pathogenesis and mechanism of MI, that is, the possible correlation of leukocytes and inflammation, has gradually become a hotspot. Previous literatures suggested that complicated and multifaceted pathologies are implicated in the MI development. In our present study, GSEA analysis showed that chemokine signaling pathway was positively associated with MI. Chemokines are originally discovered as inducing factors for leukocyte migration to the site of inflammation [23]. In addition, chemokines and their receptors also can affect homeostasis, the formation of foam cell, leukocyte activation, and so on [24]. Assisted by the bioinformatics analyses, we obtained two common genes (*CXCL16* and *NCF1*) that were both linked with chemokine signaling pathway and significantly increased in MI tissues. Ludwig et al. discovered that the vast majority of chemokines are soluble, while CXCL16 is a unique molecule, both in membrane-bound form and in soluble form [10]. Previous studies of CXCL16 in cardiovascular disease mainly focus on atherosclerosis. The soluble form of CXCL16 could promote inflammation and atherosclerosis via recruiting the inflammatory cells [25]. Furthermore, CXCL16 mRNA expression is upregulated in atherosclerotic plaques and mainly expresses in lipid-rich macrophages [26]. More importantly, CXCL16 plays an important role in the progression of heart failure through controlling the matrix remodeling [27]. CXCL16 has been reported to provide novel information in clinical cardiovascular risk assessment [28]. Xu et al. provided the first evidence that *CXCL16* polymorphisms remarkably affect MI risk in China [29]. However, its specific significance has not been verified in MI. Thus, we finally selected *CXCL16* as the topic project in this work. We analyzed the expression of CXCL16 and found that CXCL16 was significantly increased in MI tissues and the MI cell model. CCK-8 assay showed that CXCL16 inhibited cell viability of cardiomyocytes treated by H/R. These data suggested that CXCL16 might act as a vital regulator in MI development.

Considering the central role of *miRNAs* in heart diseases, we next used the TargetScan and other predicted tools to screen the upstream *miRNAs* of *CXCL16* in MI. According to the bioinformatics analysis, *miR-545* was selected as a putative upstream molecule of *CXCL16*. At present, accumulating evidences have demonstrated that *miR-545* exerts an inhibitory effect in a variety of cancers, including gastric cancer [30], hepatocellular carcinoma [20], and cervical cancer [31]. Although several *miRNAs* have been found to be involved in the development of MI via regulating the inflammatory responses after MI, such as *miR-370* [32], *miR-155* [33], and *miR-223* [34], there exist no reports of *miR-545*’s function in MI. Thus, we accessed to GEO database and displayed that *miR-545* was markedly decreased in MI tissue samples compared with normal cases, which was opposite with CXCL16 expression. Moreover, the expression of CXCL16 was attenuated by *miR-545* in the H/R cell model. Loss/gain-of-functional in vitro experiments showed that *miR-545* can partially eliminate the effect of CXCL16 on cell viability and apoptosis. PCNA, the most widely utilized proliferation labeling protein, is expressed in the nucleus and closely related with cell cycle [35]. The expression of PCNA detected by western blot confirmed that the regulation of CXCL16 in MI was associated with *miR-545*. Additionally, the primary receptor for CXCL16 is CXCR6. The CXCL16/CXCR6 axis could drive the cross-talk among cells within the brain and microglia, as well as infiltrating macrophages, and induce a neuroprotective or detrimental phenotype [36]. Mir et al. have determined the biological significance of the CXCL16/CXCR6 axis in lung cancer [37]. A controversial role of the CXCL16/CXCR6 axis was found in atherosclerosis [38, 39], suggesting that there exists a complex molecular mechanism between CXCL16/CXCR6. Maybe, this contradictory can be explained with *miR-545* in MI.

The innate immune system-induced inflammatory responses exert a crucial role in the cardiac pathological process after MI. The inflammatory responses can be triggered by MI; consequently, the release of cytokines TNF-α encourages the leukocyte infiltration and promotes the secretion of inflammatory cytokines, including IL-6 and IL-1β. These inflammatory cytokines next induce the inflammation cascade followed by myocardial dysfunction [40–43]. Levels of pro-inflammatory cytokines (TNF-α, IL-6, and IL-1β) and anti-inflammatory cytokine IL-10 modulate homeostasis within the heart in response to damage. Thus, in order to detect the role of CXCL16/miR-545 on inflammatory responses post-MI, the levels of TNF-α, IL-6, IL-1β, and IL-10 were measured using ELISA assay. Compared with the H/R group, CXCL16 resulted in a reduction in the levels of TNF-α, IL-6, and IL-1β and in an obvious increase of IL-10. By contrast, *miR-545* exerted the opposite role on the levels of inflammatory cytokines. The activity of LDH, the important marker for cellular damage [44], showed the same trend as the inflammatory cytokines, that is, CXCL16 may play a pro-inflammatory role in MI. This is consistent with a previous report, which suggests that upregulation of CXCL16 in neonatal rat cardiomyocytes can stimulate inflammation by increasing IL-1β [27]. However, Lepore et al. previously demonstrated that CXCL16 can function as a good target to modulate microglia phenotype in order to inhibit inflammation and glioma progression [36]. The opposite effects indicate that the role of CXCL16 in different diseases may be correlated with the cellular microenvironment, and its exact mechanism in MI is worthy of further investigation.

This investigation presents some limitations; for example, all results were collected from in vitro experiments, and thus, further in vivo experiments are required to validate the current observations. It is difficult to determine the clinical significance of *miR-545* and CXCL16 in actual MI patient samples. To solve this problem, we are trying our best collecting the MI patient specimens. Actually, based on the bioinformatics analysis, we discovered two upstream molecules of CXCL16, *miR-545* and *miR-34a*. The relationship between *miR-34a* and CXCL16 will be detected in another research in the future, as well as the mechanism of action of CXCL16 in the inflammatory pattern.

## Conclusion

In conclusion, our findings demonstrated that *miR-545* directly regulates CXCL16 and eliminates the injury induced by H/R stimulation via elevating cell viability and suppressing apoptosis as well as inflammatory responses. These findings might provide a novel therapeutic target for the arrangement of MI treatment.

## Supplementary Information

**Additional file 1: Table S1.** KEGG enrichment analysis based on David database (logFC≥0.6).

**Additional file 2: Table S2.** Chemokine-related genes, P = 0.016, FDR = 0.155. (XLS 2 kb)

**Additional file 3: Table S3.** Differentially expressed genes (DEGs), P = 0.016, FDR = 0.155.

**Additional file 4.**

## Data Availability

All data generated or analyzed during this study are included in this published article.

## Abbreviations

DMEM: Dulbecco’s modified Eagle’s medium
ECL: Enhanced chemiluminescence
ELISA: Enzyme-linked immunosorbent
FBS: Fetal bovine serum
GEO: Gene Expression Omnibus
GSEA: Gene Set Enrichment Analysis
H/R: Hypoxia/reoxygenation
LDH: Lactate dehydrogenase
MI: Myocardial infarction
OD: Optical density
PMSF: Phenylmethyl sulfonylfluoride
RIPA: Radio immunoprecipitation
